# Common Parasite With Uncommon Associations

**DOI:** 10.4084/MJHID.2011.015

**Published:** 2011-03-29

**Authors:** Sonal Jain, Monica Sharma, Jasmita, Seema Tyagi

**Affiliations:** Department of Hematology, All India Institute of Medical Sciences, New Delhi

**Dear Editor,**

Filaria is a common problem in India. The infection is seen endemically in the coastal regions especially around Orissa and Bihar.^1^ Incidental filariasis has previously been described in cytology smears from a wide variety of sites^1^ and its presence in a bone marrow aspirate was described as early as 1976.^2^ However, association with hematologic malignancies is limited to only an occasional case report.^3^ We are describing three cases of filaria infection detected first when the patients were investigated for a suspected hematologic disorder.

## Case 1

**Figure 1:**
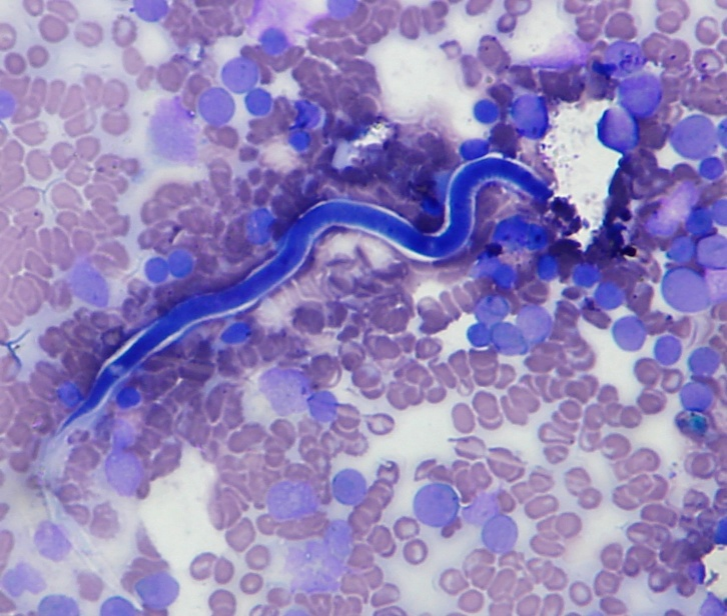
Sudan black stained bone marrow smear showing microfilaria of Wuchereria Bancrofti and SB negative blasts (400X).

A 41 year old male presented with complaints of weakness and easy fatiguablity for one year with fever for 1 month and petechial rash for 15 days. On examination, he was pale, febrile with a just palpable spleen. There was no lymphadenopathy or hepatomegaly. His hemoglobin was 12 g/dl, total leucocyte count was 98.6X10^9^/L with a platelet count of 56X10^9^/L. Differential leukocyte count on peripheral smear showed 94% blasts and a bone marrow examination was advised. The bone marrow smears showed 95% blasts which were negative for Myeloperoxidase (MPO), Sudan Black (SB), Periodic acid schiff (PAS). An occasional microfilaria was identified on the SB stained slide which was sheathed with a free cephalic space and tail tip free of nuclei. On the basis of these findings, the species was identified as Wuchereria bancrofti (Figure 1). On immunophenotyping, the blasts were positive for CD19, CD10, cytoplasmic CD79a, TdT, CD34, HLA-DR and negative for CD2, CD7, CD13, CD33, CD117, CD 64 and MPO. He was diagnosed as CD10 positive B-ALL. Following this, the peripheral smear was reviewed and it showed an occasional microfilaria. The patient was given a course of diethylcarbamazine before chemotherapy was started. He is presently on induction chemotherapy and doing well.

## Case 2

A 71 year old male presented with generalized lymphadenopathy. His cervical lymph node biopsy was suggestive of Classical Hodgkin’s lymphoma. A Staging bone marrow biopsy was performed. Hemogram showed a hemoglobin of 8.2 g/dl, TLC of 3.6 X10^9^/L with a platelet count of 84 X10^9^/L. His peripheral smear showed pancytopenia. The differential count showed 65% neutrophils, 30% lymphocytes, 3% monocytes and 2% eosinophils. There was an occasional microfilaria of Wuchereria bancrofti. Bone marrow aspirate and biopsy was cellular and did not show any evidence of lymphoma involvement.

## Case 3

**Figure 2:**
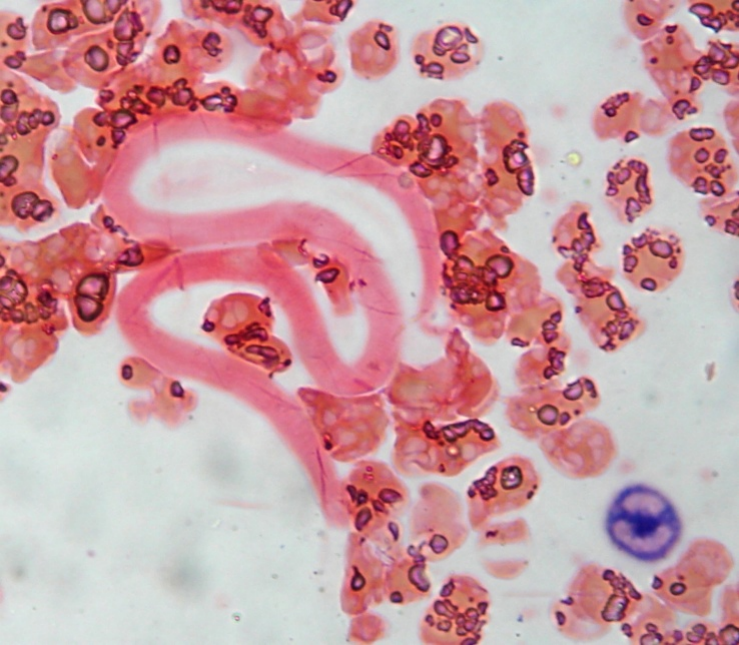
Neutrophil Alkaline Phosphatase stained peripheral smear showing microfilaria of Wuchereria Bancrofti and NAP positive neutrophil (1000X).

A 40 year old male presented to the department of hematology in view of leukocytosis. He was a case of acute onset quadriparesis with respiratory and bulbar palsy. His CECT spine was suggestive of myelitis of tubercular/ viral etiology. He was provisionally started on antitubercular therapy. His routine hemogram showed a TLC of 21.3 X10^9^/L, hemoglobin of 11.4g/dl and platelet count of 356 X10^9^/L. in view of leucocytosis, a NAP score was advised. The NAP score was 185 with a control of 162. Incidentally on the NAP stain, a few microfilariae were seen (Figure 2). A repeat hemogram and peripheral smear examination were done which showed neutrophilic leucocytosis (N90L6E1M3) and occasional microfilariae of Wuchereria bancrofti were identified. However, there was no evidence of any hematological disease.

## Discussion

Filarial infection is endemic in some parts of India. It may be associated with any benign or malignant, local or systemic disorder. Even when not suspected clinically, they may be found in peripheral smear or bone marrow smear without altering the morphological profile. The first case was a B-ALL with associated W. bancrofti infection and in the second case W. bancrofti infection was an incidental finding in a case of Hodgkin’s lymphoma. Both these patients required directed antifilarial therapy in addition to the chemotherapy. The third case highlights that parasites may be seen as incidental findings on any stain and one needs to be alert to the appearance even in special stains. In all three cases, filariasis was not suspected clinically. Although microfilariae have been detected in the bone marrow smears earlier,^2^ the first case that described their occurrence in association with acute leukemia was described by Sharma et al in 2010.^3^ Their case was AML-M4 with eosinophilia. In contrast, all three of our cases lacked eosinophilia and in one of them, we detected microfilariae on the bone marrow smears. The presence of an immunocompromised state in patients with hematological malignancies (2 of the three cases) and tuberculosis may contribute to a higher infection rate including filarial infestation. However, considering the high endemicity in our country, the filarial infestation in these cases may just be a random simultaneous occurrence.

We want to assert that parasites may be present in association with hematological malignancies and thus should always be kept in mind. This will ensure that in the presence of a more glaring malignancy, an innocuous and treatable infection is not missed. They can be detected on a special stain as well and thus, the hematopathologists should be alert to look for parasites in endemic areas in unusual situations and lack of typical associations.
